# Plasma microRNAs reproducibly identify a schizophrenia subgroup characterized by high inflammation, and associate symptom severity with inflammation

**DOI:** 10.1192/j.eurpsy.2026.11862

**Published:** 2026-07-31

**Authors:** T. Miyano, M. Hirouchi, N. Yoshimura, K. Hattori, T. Mikkaichi, N. Kiyosawa

**Affiliations:** 1 Translational Science Department II, Daiichi Sankyo Co., Ltd.; 2 Department of Psychiatry, National Center Hospital; 3 Department of Bioresources, Medical Genome Center, National Center of Neurology and Psychiatry, Tokyo, Japan

## Abstract

**Introduction:**

Schizophrenia is a complex and heterogeneous psychiatric disorder. Our previous study identified three patient subgroups characterized by different inflammation levels, defined by 40 plasma microRNAs (miRNAs) (Miyano et al. Int J Mol Sci 2024; 25 4291).

**Objectives:**

This study aimed (1) to evaluate the reproducibility of miRNA-based stratification of patients with schizophrenia, and (2) to investigate biological pathways linking symptom scores with inflammation through plasma miRNA analyses.

**Methods:**

Plasma levels of 376 miRNAs and the Positive and Negative Syndrome Scale (PANSS) were obtained in 70 schizophrenia patients. This cohort differed from the previous study in race, symptom phase, medication protocols, and the platform used for miRNA quantification. For aim (1), we examined whether the 40 previously identified miRNAs could classify three subgroups. For aim (2), we developed regression models to determine miRNAs associated with PANSS. miRNA combinations were optimized to minimize cross-validation error. We conducted a systematic literature review to evaluate whether symptom-related miRNAs exert stimulatory or inhibitory effects on pro-inflammatory cytokine expression. The study was approved by the Ethical Research Practice Committee of Daiichi Sankyo Co., Ltd.

**Results:**

The plasma miRNA profiles identified similar subgroups of patients as in the previous study, suggesting miRNA-based patient stratification is potentially reproducible. Development of regression models identified optimal combinations of 32 and 42 miRNAs for estimating the PANSS positive and negative subscales, respectively. Our literature-based survey confirmed that a part of these miRNAs exert either positive or negative effects on IL-1β, IL-6, and TNFα. Integrating the directions of the regression coefficients with the reported effects of these miRNAs on pro-inflammatory cytokines suggests that more severe positive and negative symptoms are associated with higher levels of inflammation.

**Conclusions:**

Plasma miRNAs may serve as robust biomarkers for identifying subgroups with distinct pathophysiological profiles such as inflammation. Our finding suggest that anti-inflammatory interventions may improve positive and negative symptoms in patients with high inflammation.

**Disclosure of Interest:**

T. Miyano Employee of: Daiichi Sankyo Co., Ltd., M. Hirouchi Employee of: Daiichi Sankyo Co., Ltd., N. Yoshimura: None Declared, K. Hattori: None Declared, T. Mikkaichi Employee of: Daiichi Sankyo Co., Ltd., N. Kiyosawa Employee of: Daiichi Sankyo Co., Ltd.

